# Supplementary material for: The causal role of circulating inflammatory markers in osteoporosis: a bidirectional Mendelian randomized study
Source: Front Immunol. 2024 Jul 18;15:1412298. doi: 10.3389/fimmu.2024.1412298 (PMC11291241; doi:10.3389/fimmu.2024.1412298)
Supplement: Supplementary file 1 [file DataSheet_1.zip › supplementary material/CIM-OSTEOPOROSIS /GCST90274791.csv_forest.pdf]

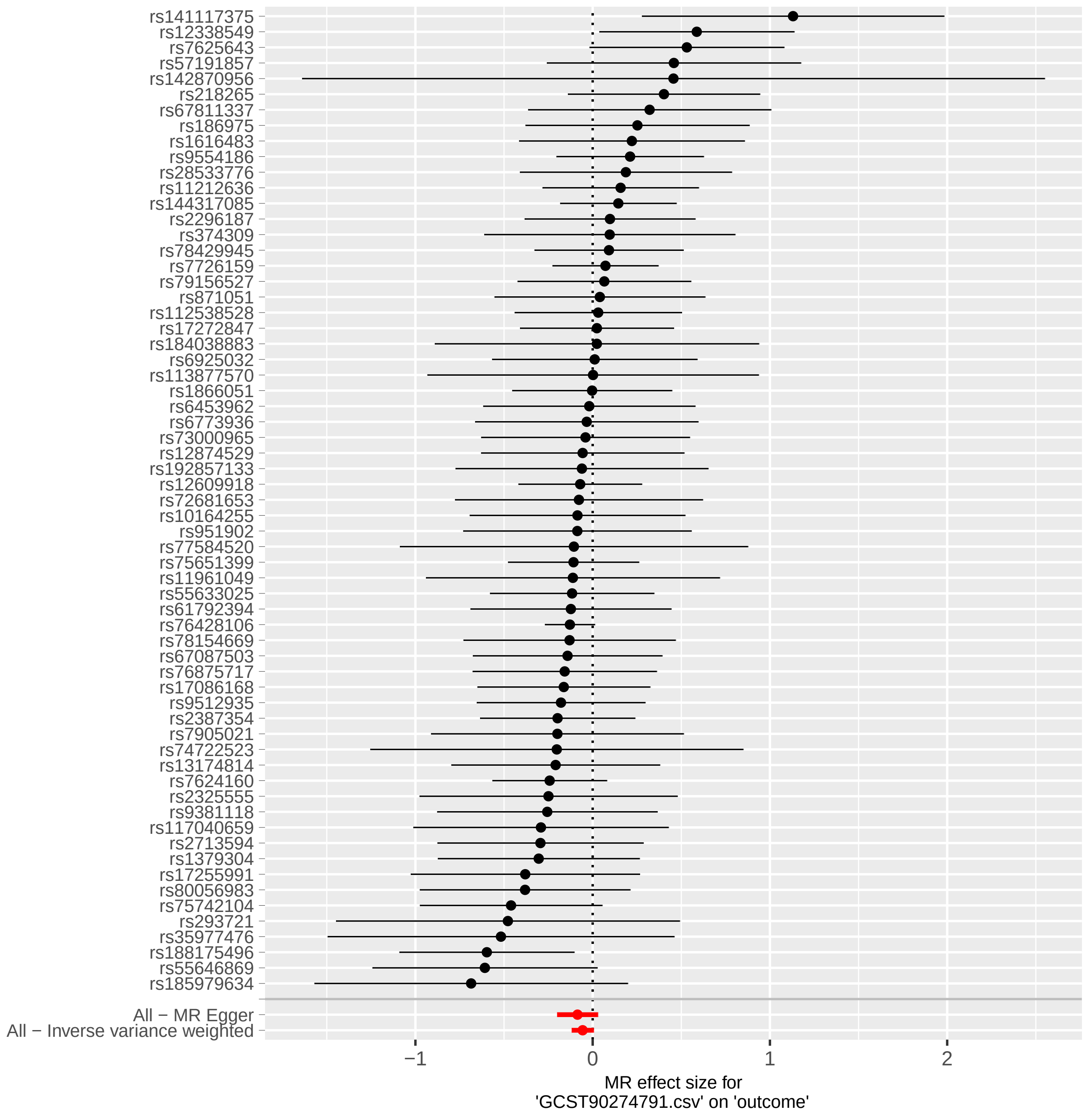
rs141117375
rs12338549
rs7625643
rs57191857
rs142870956
rs218265
rs67811337
rs186975
rs1616483
rs9554186
rs28533776
rs11212636
rs144317085
rs2296187
rs374309
rs78429945
rs7726159
rs79156527
rs871051
rs112538528
rs17272847
rs184038883
rs6925032
rs113877570
rs1866051
rs6453962
rs6773936
rs73000965
rs12874529
rs192857133
rs12609918
rs72681653
rs10164255
rs951902
rs77584520
rs75651399
rs11961049
rs55633025
rs61792394
rs76428106
rs78154669
rs67087503
rs76875717
rs17086168
rs9512935
rs2387354
rs7905021
rs74722523
rs13174814
rs7624160
rs2325555
rs9381118
rs117040659
rs2713594
rs1379304
rs17255991
rs80056983
rs75742104
rs293721
rs35977476
rs188175496
rs55646869
rs185979634
All − MR Egger
All − Inverse variance weighted
−1
0
1
2
MR effect size for
'GCST90274791.csv' on 'outcome'
